# N6-methyladenosine regulators-related immune genes enable predict graft loss and discriminate T-cell mediate rejection in kidney transplantation biopsies for cause

**DOI:** 10.3389/fimmu.2022.1039013

**Published:** 2022-11-22

**Authors:** Qidan Pang, Hong Chen, Hang Wu, Yong Wang, Changyong An, Suhe Lai, Jia Xu, Ruiqiong Wang, Juan Zhou, Hanyu Xiao

**Affiliations:** ^1^ Department of Nephrology, Bishan Hospital of Chongqing Medical University, Chongqing, China; ^2^ Department of General Surgery/Gastrointestinal Surgery, Bishan Hospital of Chongqing Medical University, Chongqing, China

**Keywords:** N6-methyladenosine (m6A), kidney transplantation, alloimmunity, graft loss, T-cell mediate rejection, biopsies for cause, prediction model

## Abstract

**Objective:**

The role of m6A modification in kidney transplant-associated immunity, especially in alloimmunity, still remains unknown. This study aims to explore the potential value of m6A-related immune genes in predicting graft loss and diagnosing T cell mediated rejection (TCMR), as well as the possible role they play in renal graft dysfunction.

**Methods:**

Renal transplant-related cohorts and transcript expression data were obtained from the GEO database. First, we conducted correlation analysis in the discovery cohort to identify the m6A-related immune genes. Then, lasso regression and random forest were used respectively to build prediction models in the prognosis and diagnosis cohort, to predict graft loss and discriminate TCMR in dysfunctional renal grafts. Connectivity map (CMap) analysis was applied to identify potential therapeutic compounds for TCMR.

**Results:**

The prognostic prediction model effectively predicts the prognosis and survival of renal grafts with clinical indications (*P*< 0.001) and applies to both rejection and non-rejection situations. The diagnostic prediction model discriminates TCMR in dysfunctional renal grafts with high accuracy (area under curve = 0.891). Meanwhile, the classifier score of the diagnostic model, as a continuity index, is positively correlated with the severity of main pathological injuries of TCMR. Furthermore, it is found that METTL3, FTO, WATP, and RBM15 are likely to play a pivotal part in the regulation of immune response in TCMR. By CMap analysis, several small molecular compounds are found to be able to reverse TCMR including fenoldopam, dextromethorphan, and so on.

**Conclusions:**

Together, our findings explore the value of m6A-related immune genes in predicting the prognosis of renal grafts and diagnosis of TCMR.

## Introduction

As of now, kidney transplantation is still the most effective remedy for end-stage renal disease (1). However, transplant patients are still chronically challenged by graft rejection, infection, and recurrence of primary kidney disease, which may lead to allograft injury and dysfunction (2). Elevated serum creatinine, hematuria, proteinuria, and decreased urine output are the common clinical manifestations of allograft injury and dysfunction. When a kidney transplant recipient develops those indications, a biopsy for cause is usually needed to identify the pathogenesis, which is known to be the gold standard (3). Timely and targeted interventions may reverse active injuries, alleviate chronic lesions, and avoid graft loss. Although the reasons for graft injury and loss are multifactorial and time-dependent, immune factors still dominate (4). Two studies on transplant kidney histology have shown that the alloimmune processes account for 35%-64% of graft loss (5, 6), of which T cell-mediated rejection (TCMR) and antibody-mediated rejection (ABMR) are the most typical subtypes. Persistent alloimmunity can also aggravate interstitial fibrosis and tubular atrophy (IF/TA), which is regarded as an important prognostic factor of grafts and the final pathological outcome of graft injuries (4).

N6-methyladenosine (m6A) modification is one of the most prevalent and reversible modifications of RNA base in eukaryotes (7). Through three functional protease complexes: writers, erasers, and readers, m6A regulates RNA transport, export, splicing, localization, translation and stability at the post-transcriptional level, thus participating in various physiological and pathological processes. Recent studies show that the m6A modification plays an important role in shaping a balanced immune response (8). M6A can affect innate, adaptive and antiviral immune responses by modulating the mRNA of key genes in the immune pathway. For example, m6A-mediated degradation of interferon B (IFNB) transcripts weakens the type I interferon and antiviral innate immune responses (9). The m6A mechanism enhances the interleukin-STAT5 signaling pathway through the attenuation of SOCS mRNA, thereby promoting the proliferation of CD4+ T cells and the immunosuppressive function of Treg cells (10, 11). M6A methylation of the Tcf7 gene mediated by METTL3 stabilizes the transcripts of the Tcf7 gene and increases the expression of TCF-1. TCF-1 promotes the differentiation of T-helper and Tfh cells, thus facilitating B cell differentiation and plasma generation (12). The regulatory role of m6A in the immune system has been demonstrated to play a part in the tumor immune microenvironment (13) and many autoimmune diseases (14), including systemic lupus erythematosus, rheumatoid arthritis, and inflammatory bowel disease. However, there is no research to elucidate its role in the immune responses after kidney transplantation.

The maturity and reduced cost of sequencing technology have improved the accuracy of disease diagnosis and treatment. Genome-wide transcript microarray data can be derived from a morsel of graft tissue, which makes it feasible for us to explore the internal relations of diseases at a molecular level. The combined application of transcript data with machine algorithms has brought about a range of molecular classifiers and risk scoring models that facilitate diagnosis and predict prognosis. Histologic diagnosis is flawed by subjective interpretations among pathologists, nonspecific lesions, and arbitrary rules, making it not as reliable as we expect (15, 16). Given the absence of a reliable gold standard, classification criteria based on objective molecular expression data present an alternative approach and complement the histologic diagnosis. Reeve et al. (17) established the Molecular Microscope Diagnostic System (MMDx) based on microarray gene expression data of renal grafts, whose balanced accuracies for histology diagnoses of TCMR and ABMR reach 73% and 78%, respectively. The molecular risk score established by Einecke et al. (18) is able to reflect active injury and superior to either scarring or function in predicting graft failure.

This study aims to explore the relations between m6A modification and immune factors behind renal graft injury at the molecular level by analyzing the microarray data of kidney transplantation biopsies for cause. By analyzing the gene expression data of discovery cohort, we found that m6A regulators are closely related to a variety of immune characteristics, which are mainly involved in alloimmune processes and T cell subsets, suggesting the unique value of m6A modifications in TCMR. Based on machine learning, we managed to build a risk score and a molecular classifier to predict graft outcomes and distinguish TCMR from other types of graft injury, respectively. In short, our findings suggest that m6A modification is involved in graft dysfunction after transplantation by regulating the immune response and provides a reference for subsequent studies.

## Materials and method

### Collection and processing of data

The microarray expression data used in this study were derived from research accession published in the Gene Expression Omnibus (GEO, https://www.ncbi.nlm.nih.gov/geo/) database. The inclusion criteria included: (1) consecutive cohort; (2) samples derived from kidney biopsies for clinical indications; and (3) including TCMR and ABMR pathologic diagnosis based on Banff criteria or graft survival data. We managed to screen out 4 datasets, of which GSE360591 (19) was used as the discovery cohort, GSE213742 (18) prognosis cohort, and GSE485813 (20) and GSE983204 (21) diagnosis cohorts. All microarray datasets were subjected to log2 transformation and normalized using the R “limma” package. Two expression matrices in the diagnosis cohort were transformed by z-score to increase the comparability between independent datasets.

### Correlation analysis of m6A regulators with immune characteristics

We identified 23 m6A regulators from the previous literature (22), including 8 writers (METTL3, METTL14, METTL16, WTAP, VIRMA, ZC3H13, RBM15, RBM15B), 13 readers (YTHDC1, YTHDC2, YTHDF1, YTHDF2, YTHDF3, HNRNPC, FMR1, LRPPRC, HNRNPA2B1, IGFBP1, IGFBP2, IGFBP3, RBMX) and 2 erasers (FTO, ALKBH5). Characteristic gene data of 22 kinds of immune cells were collected from the CIBERSORTS (23) database (https://cibersortx.stanford.edu/), and the immune cell abundance of each sample in the discovery cohort was calculated using the CIBERSORT.R script. Immune gene ontology categories/gene sets were downloaded from the ImmPort (24) database (https://www.immport.org/), and the R “GSVA” package was used to perform the single sample gene set enrichment analysis (ssGSEA) to obtain an enrichment score for each sample based on immune gene sets. 35 key genes of allograft rejection pathway (map05330) were obtained from the Kyoto Encyclopedia of Genes and Genomes (KEGG, https://www.kegg.jp/) database, as well as the expression matrix of key genes in the cohort sample. We conducted the R cor.test () to figure out the correlation coefficient between m6A regulators gene expression and immune cell abundance, immune gene sets enrichment score as well as rejection key genes expression of samples in the cohort and the correlation heat map was plotted using the R “ggplot2” package.

### Establishment and analysis of the prognostic prediction model

First, we performed the correlation analysis between 1795 (after removing 704 duplicates) immune genes of 17 immune categories and m6A regulators in the discovery cohort. Those immune genes were derived from ImmPort database. 278 m6A-related immune genes (MRIGs) (|correlation coefficients|> 0.6 and *P*< 0.01) were obtained, on which gene enrichment analysis was conducted *via* the R “clusterProfiler” package. Then, we performed the univariate cox regression analysis between MRIGs and graft survival data, which was assessed as the time between biopsy and graft failure/censoring, and obtained 108 prognostic m6A-related immune genes (P-MRIGs), taking *P*<0.001 as the cutoff value. Finally, the R “caret” package was used to randomly divide the prognosis cohort into train cohort and test cohort, with a ratio of 1:1. In the train cohort, we carried out the least absolute shrinkage and selection operator (Lasso) regression with 10-fold cross validation on P-MRIGs using R “glmnet” package and selected the P-MRIGs corresponding to the smallest lambda value for model building. The multivariate Cox regression was conducted to figure out the regression coefficient. The Risk Score was calculated with the following formula: Risk Score = $\sum_{i=1}^{n}\left(coef_{i}*expr_{i}\right)$ , here *expr_i_* represented the expression level of gene, *i* and *coef_i_*, the regression coefficient of gene *i* in the signature. The train cohort was divided into high- and low-risk groups, choosing median of risk score as the midpoint. The Kaplan-Meier survival curve was plotted using the R “survminer” package. Log Rank test was used to compare the differences in graft survival between the two risk groups and the ROC curve drew by R “timeROC” package plots evaluated the predictive performance of the signature. Similar proceedings were carried out in the test cohort. In addition, to verify the model’s applicability, we conducted the graft survival analysis of high- and low-risk groups in the rejection group and non-rejection group respectively, ran the GSEA 4.1.0 software (25) to identify the underlying pathophysiology of the risk-group and compared the gene enrichment differences in the KEGG pathway between the high and low-risk groups.

### Differential analysis of m6A regulators and immune characteristics in subgroups

The distribution differences of m6A regulators and immune characteristics, which were obtained from the proceedings above, including gene expression matrix, the abundance of immune cells, and immune gene set enrichment scores, were compared in the TCMR, ABMR, and non-rejection groups of the discovery cohort. The results were visualized using R “pheatmap,” “ggplot2”, and “ggpubr” packages.

### Establishment and analysis of the diagnosis prediction model

We intended to build a diagnostic model of TCMR based on m6A-related immune genes. Firstly, we performed a gene differential analysis between TCMR and non-TCMR groups (including the mixed group) in the discovery cohort using R “limma” package and obtained 120 differentially expressed genes (DEGs, | Log Fc |> 1, *P*< 0.05), of which 64 DEGs are immune-related genes. Subsequently, we carried out a correlation analysis between 64 differentially expressed immune genes and m6A regulators, and further obtained 58 m6A-related immune genes (DE-MRIGs, |correlation coefficients|> 0.4 and *P*<0.05) in the diagnosis cohort (train). Cycloscape 3.8.0 software (26) was used to perform protein-protein interaction (PPI) analysis of DE-MRIGs and corresponding m6A regulators. The GlueGO pluglet (27) was used for enrichment analysis and visualization. Finally, we utilized R “randomForest” package to carry out the decision tree analysis of DE-MRIGs to select feature genes in the train cohort. The appropriate variables were selected on the basis of their importance to build the model and logistic regression was conducted to determine the variable regression coefficient. The classifier score was calculated with the following formula: Classifier Score = $\sum_{i=1}^{n}\left(coef_{i}*expr_{i}\right)$ .,here *expr_i_* represented the expression level of gene, *i* and *coef_i_*,the regression coefficient of gene *i* in the classifier. The diagnostic performance of classifier was evaluated by the Area Under Curve (AUC) of the ROC Curve. The Optimal cut-off point was determined based on Youden index. Classifier score was calculated and evaluated in the test cohort. Moreover, a violin plot was drawn based on the histological lesions in the test cohort to compare the distribution of classifier score in TCMR-related injuries of different degrees, in which Wilcoxon Rank Sum was used for comparison between two groups, Kruskal-Wallis test for comparison between multiple groups. We retrieved Connectivity Map (Cmap) Database (https://clue.io/) (28) to identify the potential compounds that could alleviate TCMR lesions. Potential drugs with absolute Cmap score over 95 were selected and visualized using the R ComplexHeatmap package.

## Results

### Characteristic of cohort and biopsy

A total of 4 consecutive study cohorts, including 2193 renal transplant biopsy samples and 1906 kidney transplant patients, are included in this study. The detailed information is shown in **Table 1**. 36%-62% of renal graft biopsies are performed due to rapid or slow deterioration of graft function. The median time of biopsy after transplantation ranges from 512 to 751 days, of which 55%-100% of renal allografts biopsies one year after transplantation. Biopsies with a definite pathological diagnosis or lesions associated with alloimmunity, including TCMR, ABMR, mixed ABMR and TCMR, borderline rejection, and transplant glomerulopathy (TG) are most common (24%-48%), among which TCMR and ABMR have similar incidence. 55%-83% of transplant patients are given maintenance immunosuppressive regimens, which include calcineurin inhibitors at the time of biopsy. 12%-29% of recipients undergo graft failure, with mean follow-up time after transplantation ranging from 469 to 1017days.

**Table 1:** Characteristic at cohort and biopsy.

GEO accession	GSE36059	GSE21374	GSE48581	GSE98320
**Cohort type in study**	discovery cohort	prognosis cohort	diagnosis cohort (train)	diagnosis cohort (test)
**Platform**	GPL570	GPL570	GPL570	GPL15207
**Sample tissue**	kidney transplant biopsies	kidney transplant biopsies	kidney transplant biopsies	kidney transplant biopsies
**Sample size**	403	105/282*	300	1208
**number of patients**	315	105	264	1045
**Indication for biopsy**				
Primary nonfunction(including DGF)	10 (2%)	unknown	9 (3%)	53 (5%)
Deterioration of graft function	246 (61%)	65 (62%)	170 (57%)	436 (36%)
Stable impaired graft function	71 (18%)	7 (7%)	17 (6%)	79 (7%)
Investigate proteinuria/rejection/BK/creatinine	38 (9%)	15 (14%)	71 (24%)	175 (14%)
Follow-up from previous biopsy	unknown	6 (6%)	unknown	unknown
Others	23 (6%)	6 (6%)	17 (6%)	443 (37%)
Indication unknown	15 (4%)	6 (6%)	16 (5%)	22 (2%)
**Time of biopsy after transplant (d)**				
mean time	1437	1734	1705	unknown
median time (range)	512 (6-12831)	unknown	751 (3-9889)	591 (1-11453)
Early biopsies (< 1 year)	182 (45%)	0 (0%)	116 (39%)	507 (42%)
Late biopsies (≥ 1 year)	221 (55%)	100 (100%)	184 (61%)	701 (58%)
**Diagnosis (conclusive)**				
TCMR	32 (11%)	14 (13%)	35 (9%)	87 (7%)
ABMR	40 (13%)	11 (10%)	65 (16%)	24 (2%)
Mixed ABMR and TCMR	6 (2%)	3 (3%)	22 (5%)	41 (3%)
Borderline rejection	46 (15%)	11 (10%)	42 (10%)	109 (9%)
Transplant glomerulopathy(TG)	20 (7%)	unknown	4 (1%)	40 (3%)
Glomerulonephritis	40 (40%)	22 (21%)	41 (10%)	97 (8%)
BK virus	13 (4%)	1 (1%)	13 (4%)	37 (3%)
No major abnormalities	43 (14%)	unknown	76 (19%)	274 (23%)
**Maintenance immunosuppression at biopsy (calcineurin inhibitors)**				
Tacrolimus	176 (44%)	38 (36%)	127 (42%)	712 (59%)
Cyclosporine	101 (25%)	49 (47%)	38 (13%)	192 (16%)
**Time of follow-up after biopsy (d, mean time)**	1017	774	469	unknown
**Failed grafts**	80 (25%)	30 (29%)	33 (12%)	unknown

*GSE21374 provided a total of 282 samples, but was only able to find histological information for 105 of them.

### Correlation between m6A regulators and immune characteristics

**Figure 1:**
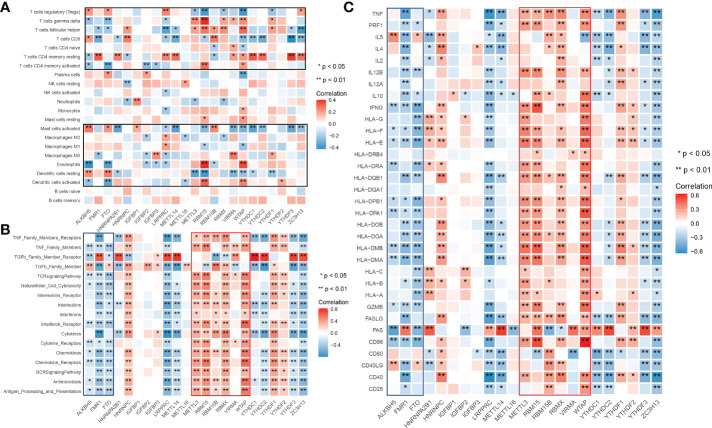
Landscape of correlationship between m6A regulators and immune characteristics. **(A)** Correlation heatmap of m6A regulators and immune cells. **(B)** Correlation heatmap of m6A regulators and immune gene categories. **(C)** Correlation heatmap of m6A regulators and rejection key genes. m6A regulators significantly correlated immune cells clustered in the black wireframe; Immune gene categories or rejection key genes significantly positive correlated m6A regulators clustered in the red wireframe; Immune gene categories or rejection key genes significantly negative correlated m6A regulators clustered in the blue wireframe.

To explore whether m6A is related to immune factors, especially alloimmunity at the molecular level in the process of graft dysfunction, we collected and processed the data with immune characteristics and performed the correlation analysis with m6A regulators. Most m6A regulators are significantly correlated with T cell subtypes, macrophages, dendritic cells, mast cells and eosinophils, but not B cells (**Figure 1A**). Similar findings are observed in the respective correlation analysis of m6A regulators and immune categories, as well as m6A regulators and rejection key genes, as shown in **Figures 1B, C**. Erasers are mainly negatively related to the corresponding immune characteristics (shown in blue wireframe) while writers are mainly positively related (shown in red wireframe). For readers, both positive and negative correlations can be found, which may be related to its property of adjustment.

### M6A-related immune gene-based prognostic prediction model for graft loss

**Figure 2:**
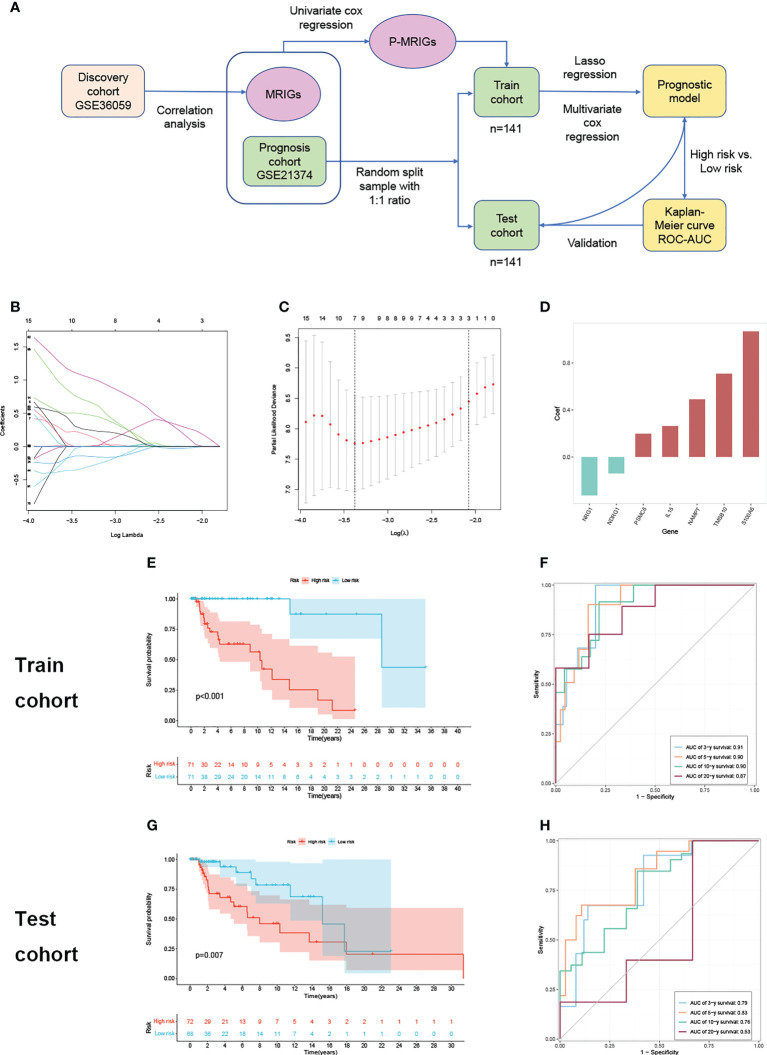
Construction and verification of prognostic prediction model. **(A)** Flow of constructing the prognostic prediction model. **(B)** Lasso coefficient profiles. **(C)** The partial likelihood deviance plot. **(D)** Coefficient of seven screened P-MRIGs in the prognostic prediction model. **(E)** The K-M curve showed that the high-risk group had a more inferior graft survival than the low-risk group in train set and **(G)** test set. **(F)** ROC curve of the model: the AUCs of 3-, 5-,10- and 20-year graft survival in the train set and **(H)** test set. Lasso, least absolute shrinkage and selection operator; P-MRIGs, prognostic m6A-related immune genes; K-M, Kaplan–Meier; ROC, receiver operating characteristic; AUC, areas under the curve.

Given what we have discovered above, we assumed that m6A modification-related immune molecules may be able to affect the outcomes of renal grafts, on which we established a prognostic model of grafts. The flow of modeling is shown in **Figure 2A**. We found that most genes are enriched in T cell activation, regulation of response to biotic stimulus, cytokine receptor interaction, and PI3K-Akt signaling pathway (**Supplementary 1**) on gene set enrichment analysis of 278 m6A-related immune genes (MRIGs). A list of 108 m6A-related prognostic genes immune genes (P-MRIGs) with hazard ratios (HR) is recorded in **Supplementary 2**. Based on Lasso regression, 7 P-MRIGs (S100A6, TMSB10, NAMPT, IL15, PSMC6, NDRG1, NRG1) are determined for building the prognostic prediction model (**Figure 2D**). Each candidate gene is given a corresponding coefficient by multivariate Cox regression, and the risk score of each sample is calculated. Taking the median of risk score as the threshold, we stratified the train cohort into different risk groups, of which the graft survival probability of the high-risk group is significantly lower than that of the low-risk group (P<0.001, **Figure 2E**). The model shows good predictive performance (**Figure 2F**), as AUC for predicting graft survival of 3-year, 5 -year, 10- year, and 20-year are 0.91, 0.90, 0.90, and 0.87, respectively. The model was verified in the test cohort. Similarly, the high-risk group has remarkably poor graft survival (P< 0.007, **Figure 2G**), as AUC for predicting graft survival of 3-year, 5 -year, 10- year, and 20-year are 0.79, 0.83, 0.76, and 0.53, respectively (**Figure 2H**).

**Figure 3:**
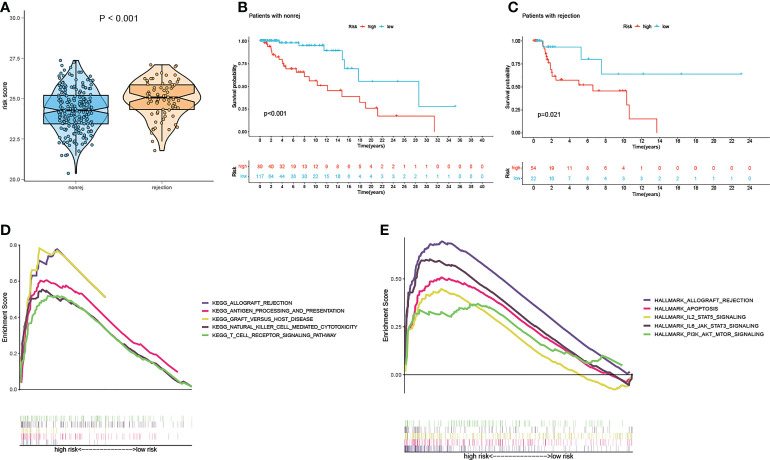
Test of suitability of the prognostic prediction model. **(A)** Violin plot of risk score in non-rejection and rejection group. **(B)** The K-M curve showed that the high-risk group had a more inferior graft survival than the low-risk group in non-rejection and **(C)** rejection group. **(D)** Gene enrichment analysis of KEGG pathway and **(E)** hallmark pathway in high-group versus low-group. K-M, Kaplan–Meier.

There is a significant difference in the distribution of risk scores between the rejection and non-rejection groups (**Figure 3A**), and the risk score is higher in the rejection group (p<0.001). Moreover, the KM survival curves show that graft survival of the high-risk group is much worse than that of the low-risk group regardless of with rejection or not (**Figures 3B, C**), which indicates that the predictive performance of the model is not affected by rejection factors and possesses of strong applicability.

In order to explore the latent causes behind the poor graft survival of the high-risk group, we compared the gene enrichment of the two risk groups. In high-risk group, more genes are enriched in the pathways related to alloimmunity, such as allograft rejection and graft versus host disease, suggesting alloimmunity is the principal element accounting for graft loss.

### M6A regulators and immune characteristics in rejection versus non-rejection

**Figure 4:**
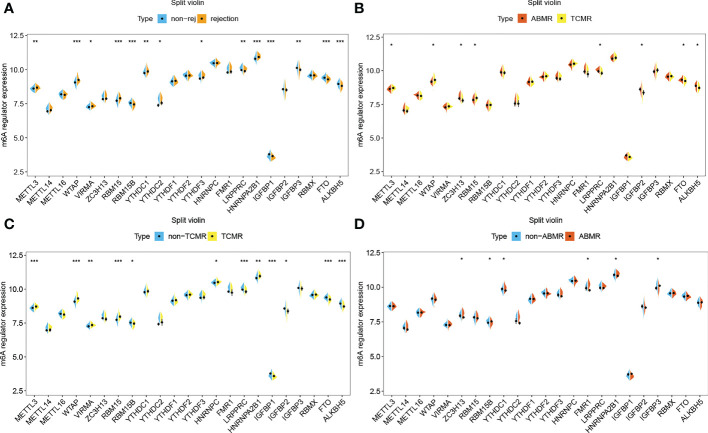
m6A regulators in rejection subtypes. **(A)** Split violin plot of m6A regulators’ gene expression levels in non-rejection versus rejection group,**(B)** ABMR versus TCMR group, **(C)** non-TCMR versus TCMR group, and **(D)** non-ABMR versus ABMR group. *P< 0.05, **P< 0.01, ***P< 0.001.

The gene expression differences of m6A regulators in the rejection group, including TCMR and ABMR, as well as non-rejection, are shown in **Figure 4**. For most of m6A regulators, their gene expression levels are significantly different between rejection group and non-rejection group (**Figure 4A**). Similarly, the expression levels of most of the m6A regulators are remarkably different between TCMR and non-TCMR groups. However, only a few m6A regulators show a significant difference in gene expression levels between ABMR and non-ABMR, as well as ABMR and TCMR (**Figures 4B, D**). Thus, we speculated that m6A regulators may play an important part in rejection, especially in TCMR, while its role in AMBR is limited.

The results of immune cell infiltration show that there are more CD4 or CD8 T cells, helper T cells, M1 macrophages, activated dendritic cells, and eosinophilia infiltrated in the TCMR group (**Supplementary 3A, B**), which are precisely the immune cell types significantly related to m6A regulators. The TCMR group has higher enrichment scores in a number of immune categories (**Supplementary 2C, D**), which are also significantly related to m6A regulators. Thus, it is justifiable to conclude that m6A-modified immune responses play a specific role in the pathogenesis of TCMR.

### M6A-related immune gene-based diagnostic prediction model for TCMR

**Figure 5:**
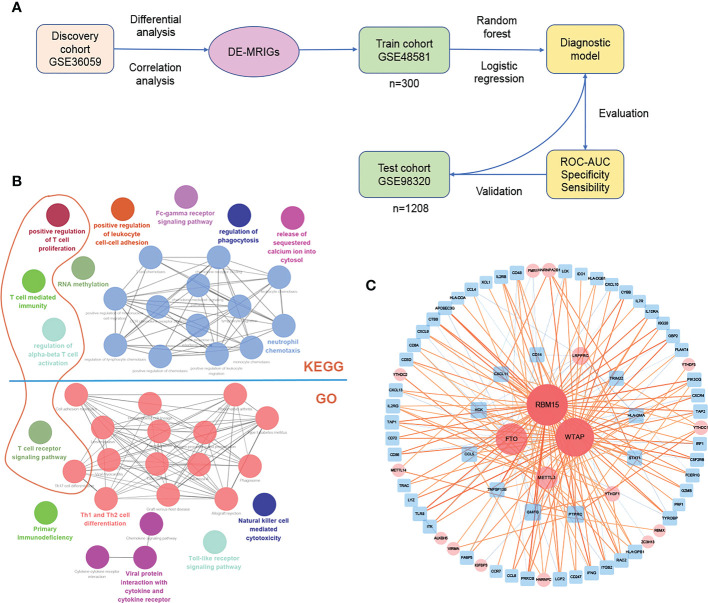
Construction and verification of diagnostic prediction model. **(A)** Flow of constructing the diagnostic prediction model. **(B)** Gene enrichment analysis and **(C)** network of DE-MRIGs and corresponding m6A regulators. DE-MRIGs, different expression m6A-related immune genes.

The process of establishing the prediction model is shown in **Figure 5A**. Several genes are enriched in T cells immunity, proliferation, and related pathway (Dark orange circles in **Figure 5B**) on the enrichment analysis of DE-MRIGs and their counterpart m6A regulators. **Figure 5C** shows the network of DE-MRIGs and m6A regulators, of which RBM15, WTAP, FTO, and METTL3 may be the hub genes that regulate the immune genes.

**Figure 6:**
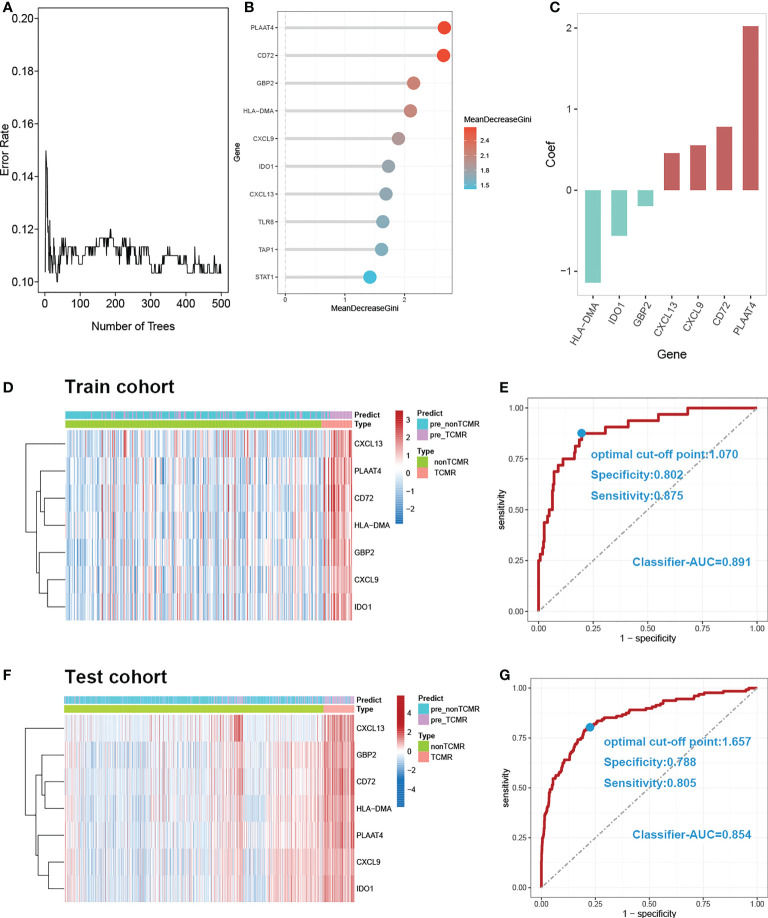
Construction and verification of diagnostic prediction model. **(A)** Random forest error rate plot. **(B)** Mean decreased gini of genes profiles. **(C)** Coefficient of seven screened DE-MRIGs in thedignostic prediction model. **(D)** Heatmap of identified DE-MRIGs in train cohort and **(F)** test cohort. **(E)** ROC curve of the model: the AUC, optimal cut-off point, specificity and sensitivity of classifier for discriminating TCMR in train cohort and **(G)** test cohort. DE-MRIGs, different expression m6A-related immune genes; ROC, receiver operating characteristic; AUC, areas under the curve.

7 DE-MRIGs with the greatest mean decrease of Gini coefficient are selected for modeling by the Random Forest algorithm (**Figure 6B**). **Figure 6A** demonstrates that when the decision trees are accumulated to a certain number, the error of the random forest model falls between 10% and 12%. The regression coefficient of each DE-MRIG was obtained by Logistics regression, and then the classifier score of each sample in the train cohort was calculated. The expression levels of 7 DE-MRIGs in the sample and their corresponding histological and predicted diagnosis types are shown in **Figure 6D**. The classifier possesses excellent predictive performance for TCMR with an AUC of 0.891. The specificity and sensitivity of the model are 80.2% and 87.5%, respectively, when the optimal cut-off point is 1.070. We also verified the model in the test cohort, and it still shows good performance with an AUC of 0.854 when the optimal cut-off point is 1.657. The model was verified in the test cohort which also delivers good performance with an AUC of 0.854, and the optimal cut-off point is 1.070, affected by sequencing platforms. The specificity and sensitivity in the test cohort are 78.8% and 80.5%, respectively.

**Figure 7:**
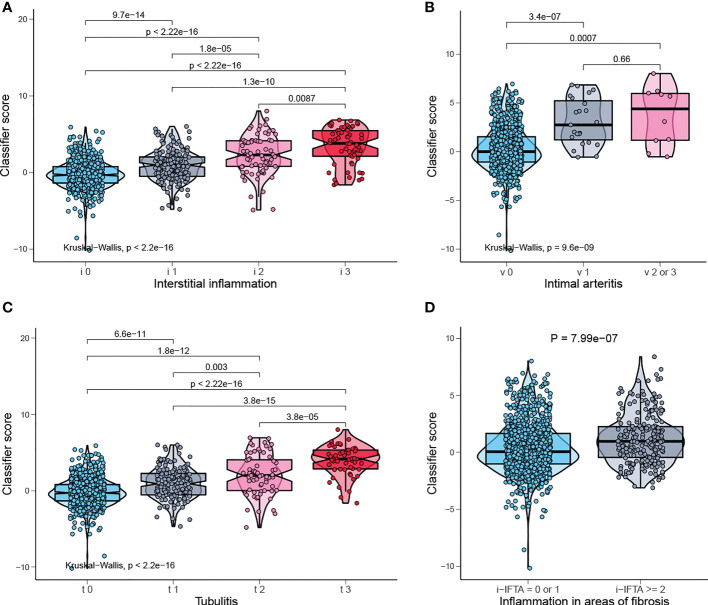
Classifier score in TCMR-related pathological lesion. **(A)** Classifier score in i0, i1, i2, i3, **(B)** v0, v1, v2 or 3, **(C)** t0, t1, t2, t3, and **(D)** i-IFTA=0 or 1, i-IFTA > =2. i, interstitial inflammation; t, tubulitis; v, intimal arteritis; i-IFTA, inflammation in areas of fibrosis.

Banff lesions i, t, v, i-IFTA represent interstitial inflammation, tubulitis, intimal arteritis, and inflammation in areas of fibrosis/interstitial fibrosis and tubular atrophy, respectively. Those are the main pathological lesions of acute and chronic TCMR, and the diagnosis is exactly based on them. The distribution of the classifier score shows a significant gradient difference in injury indicators of TCMR, which means the classifier scores increases with the degree of injury (**Figures 7A, C**). Therefore, our model can be used to reflect the severity of pathological injury and facilitate in TCMR grading.

The Connectivity Map (CMap) database can explore the potential therapeutic small molecule compounds by comparing the uploaded gene signature with the in-house gene datasets, from which the corresponding correlation score is obtained, namely the CMap score. We screened out the compounds for TCMR in the CMap database based on the signature of the top 10 DE-MRIGs (**Supplementary 4**) in the diagnostic model. The potential therapeutic drugs with absolute CMap score over 95 are selected, and the most common mechanism of action is antagonizing adenosine receptor (**Supplementary 5**).

## Discussion

The kidneys are a common target of systemic immune and autoimmune disorders, which is partly related to the size-selective and charge-dependent filtration process (29). In terms of transplanted kidneys, persistent and intense alloimmunity is the main culprit for graft loss. There is accumulating evidence suggesting new functions of m6A in regulating various aspects of immunity, including immune recognition, activation of innate and adaptive immune responses, and cell fate decisions (8). It is justifiable to speculate that m6A may also be involved in regulating alloimmunity and other immune responses in renal transplantation. Thus, we derived microarray expression data from transplanted kidney biopsies for cause and tried to explore the relations between m6A regulators and immune responses in renal transplantation at a molecular level, on which the diagnostic and prognostic models were built.

Einecke et al. (18) first reported a molecular classifier for predicting future graft loss in late kidney transplant biopsies. The transcripts that are associated with graft loss and used as a classifier, can only give us hints about tissue injury and fail to reflect the inflammatory state. Although transcripts in this research are limited to immune genes associated with m6A modifications, with an AUC of 0.9, they have better performance in predicting graft survival of 3-, 5-, and 10- years than the classifier in the previous study, whose AUC is 0.83. Moreover, our model is applicable for patients with or without rejections in predicting graft survival.

The m6A-related immune genes included in the prognostic model may also play a consequential role in the risk stratification of graft loss. S100A6 protein belongs to the S100 protein family of Ca2+- binding proteins (30). Research revealed that interferon beta (INFβ) activity could be modulated *via* the binding of S100A6 protein (31). Yilmaz et al. (32) found that NAMPT can reflect endothelial dysfunction directly following renal transplantation. In kidney transplantation, IL-15 can stimulate CD4 + CD28 null T cells to generate alloreactivity (33) or acts as a biomarker for the assessment of antibody-mediated kidney allograft rejection (34).

In the wake of new immunosuppressive regimens, TCMR is less common but still remains the dominant early rejection phenotype and serves as the endpoint for clinical trials (35). The latest Banff classification outlines the diagnostic criteria of TCMR based on four histological lesions: interstitial inflammation (i2 or i3), tubulitis (t2 or t3), intimal arteritis (v1, v2 or v3), and inflammation in areas of interstitial fibrosis and tubular atrophy (i-IFTA2 or i-IFTA3) (36). This scoring system is largely opinion-based and inconsecutive with arbitrary cutoffs (37). Moreover, the histological lesions for TCMR are nonspecific. For example, interstitial inflammation and tubulitis are also found in acute kidney injury (AKI), possibly rendering false positives, and difficult to assess in scarred tissue, causing false negatives (20). Advantages of molecular assessment over histological approaches include objectivity, repeatability and quantification, which can emerge as an amelioration to pathological diagnosis (37).

In the pathogenesis of TCMR, effector T cells, dendritic cells and activated macrophages are the main acting cells (37), which are also significantly associated with m6A regulators in the discovery cohort. At the same time, there is a remarkable difference in the expression of most of these m6A regulators between TCMR and non-TCMR, indicating that m6A modification may play a part in TCMR, on which we established a diagnostic prediction model to identify TCMR in grafts dysfunction. The classifier score of our model outperforms the published molecular test - TCMR score in diagnostic performance with an AUC of 0.89 vs 0.8412. In addition, further analysis revealed that the classifier score is positively related to the degree of main pathological lesions of TCMR, enabling it to evaluate pathological injury degree and further grade TCMR.

**Figure 8:**
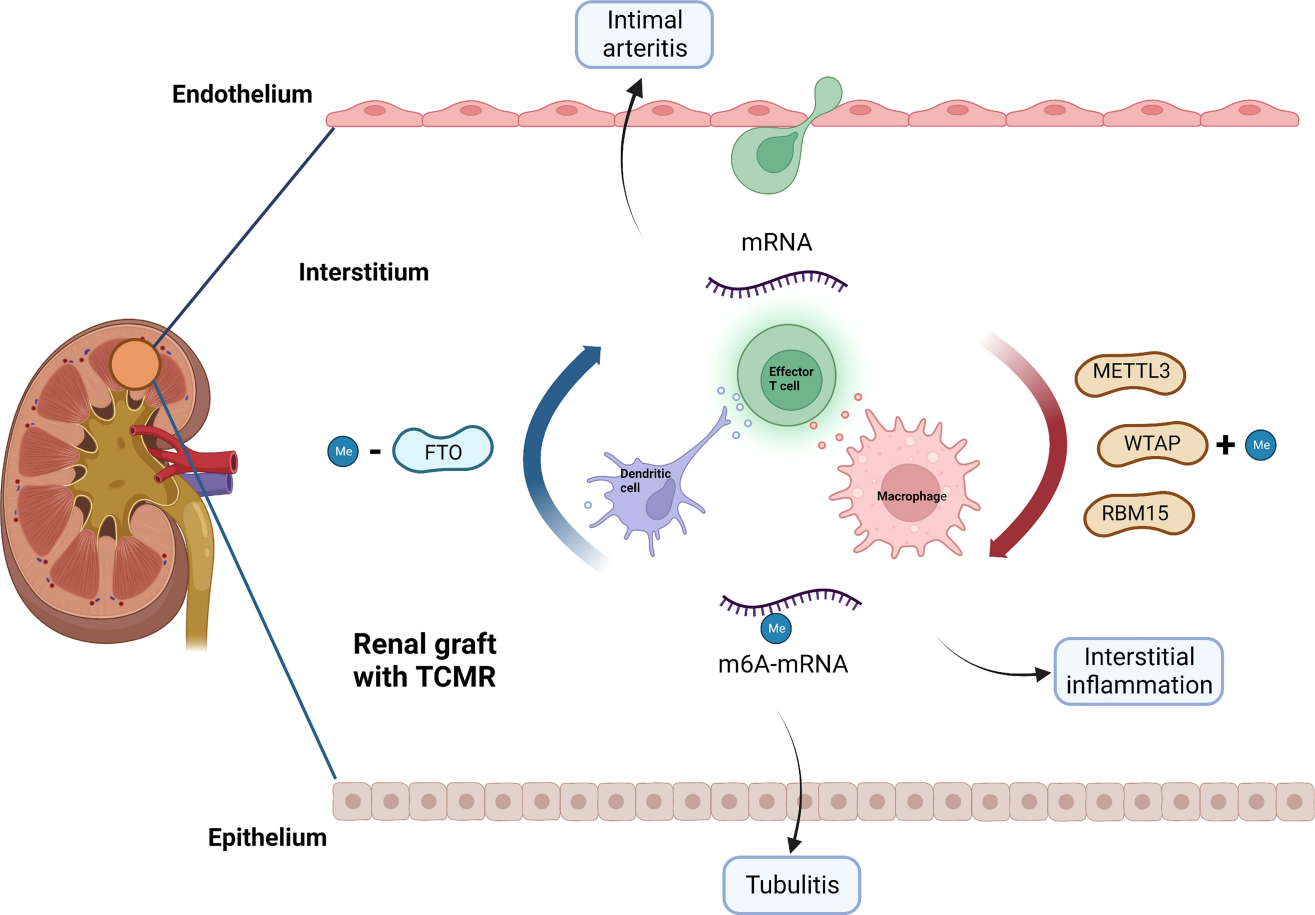
Potential role of m6A modification in TCMR. METTL3, WTAP, RBM15 and FTO may be involved in the pathogenesis of TCMR by regulating immune response, thereby causing interstitial inflammation, tubulitis and intimal arteritis. TCMR, T cell mediated rejection; Me, N6-methyladenosine.

Through network analysis, we found that METTL3, FTO, WATP and RBM15 may play a pivotal part in the regulation of immune responses in TCMR (**Figure 8**). It has been proven that METTL3 regulates T cell homeostasis (38), M1 macrophage polarization (39) and dendritic cell maturation (40); FTO enhances M1 and M2 macrophage activation (41); WTAP controls T cell receptor signaling and survival of T cells (42). It is noted that the m6A-related immune genes, which were finally screened out to build our model, namely, CD72 (43), CXCL9 (44) and CXCL13 (45), have also been reported to emerge as biomarkers for TCMR-exclusive.

Furthermore, we managed to select some small molecule compounds that may be able to reverse TCMR damage. Among the compounds with highest CMap scores, fenoldopam has been proven to be able to alleviate acute kidney injury (46) and is promising for reversing delayed graft function (DGF) (47);dextromethorphan can reduce renal complications of diabetes (48). We identified the potential therapeutic drugs with absolute CMap score over 95, and the most common mechanism of action is antagonizing adenosine receptor. Debra et al. (49) confirmed in mice experiment that adenosine receptor antagonists could protect against kidney injury.

This study has some limitations: on one hand, there is no clinically relevant population being studied, which is referred to as “limited challenge bias” (36); on the other hand, the hypothesis requires *in vivo* and vitro experiments to verify. There are also some common problems in the buildup of transcriptome models: firstly, there is inevitable inaccuracy in adopting histology based on Banff classification as the gold standard of diagnosis. Secondly, the deviation can be generated from the transcript data obtained from different experimental platforms. Due to this bias, the optimal cutoff points derived from train and test cohorts in the diagnostic model are quite discrepant in this study. Finally, for-cause biopsies are mainly performed for those patients with clinical indications. The inclusion itself has already resulted in selection bias, which may overestimate the model’s performance. Therefore, it is better to study the relations between m6A and transplant rejection in patients with protocol biopsy and diagnose rejection at an earlier stage. Anyhow, our study is pioneering and enlightening,and provides valuable clues for future studies on the role of m6A modification in renal graft dysfunction.

## Conclusion

Collectively, our findings demonstrated that m6A-related immune genes could be used for prediction of graft loss and diagnosis of TCMR, which may be involved in the process of renal graft dysfunction. The results of this study offer novel schemes for molecular assessment of disease states in kidney transplant and provide a ponderable direction for the future research.

## Data availability statement

Publicly available datasets were analyzed in this study. This loosedata can be found here: GSE360591 GSE213742 GSE485813 GSE983204.

## Author contributions

QP and HX conceived and designed the study. HX, HC, HW, YW, and CA performed the data analysis. QP wrote the original draft. JZ, SL, JX, RW, and HX reviewed and revised the manuscript. All authors contributed to the article and approved the submitted version.

## Acknowledgments

We appreciate Professor Philip F. Halloran and researchers of Alberta Transplant Applied Genomics Centre for their contribution to the molecular assessment of disease states in kidney transplant biopsy samples and their data available in the GEO database.

## Conflict of interest

The authors declare that the research was conducted in the absence of any commercial or financial relationships that could be construed as a potential conflict of interest.

## Publisher’s note

All claims expressed in this article are solely those of the authors and do not necessarily represent those of their affiliated organizations, or those of the publisher, the editors and the reviewers. Any product that may be evaluated in this article, or claim that may be made by its manufacturer, is not guaranteed or endorsed by the publisher.

## Supplementary material

Supplementary 1 Gene enrichment analysis of m6A-related immune genes. **(A)** Bubble plot of GO analysis. **(B)** Bubble plot of KEGG analysis.

Supplementary 2 Univariate Cox regression analysis revealed that the selected m6Arelated immune-genes significantly correlated with clinical prognosis.

Supplementary 3 Immune characteristics in rejection subtypes. **(A)** Heatmap and **(B)** boxplot of immune cell infiltration in non-rejection, TCMR and ABMR. **(C)** Heatmap and **(B)** boxplot of enrichment score of immune categories in non-rejection, TCMR and ABMR.

Supplementary 4 Results of CMap analysis with absolute CMap score over 95 of top 10 important m6A-related immune genes.

Supplementary 5 The mechanisms of action shared by small molecular compounds based on CMap analysis.
