# Supplementary figures and images for: N6-methyladenosine regulators-related immune genes enable predict graft loss and discriminate T-cell mediate rejection in kidney transplantation biopsies for cause
Source: Front Immunol. 2022 Nov 22;13:1039013. doi: 10.3389/fimmu.2022.1039013 (PMC9722771; doi:10.3389/fimmu.2022.1039013)

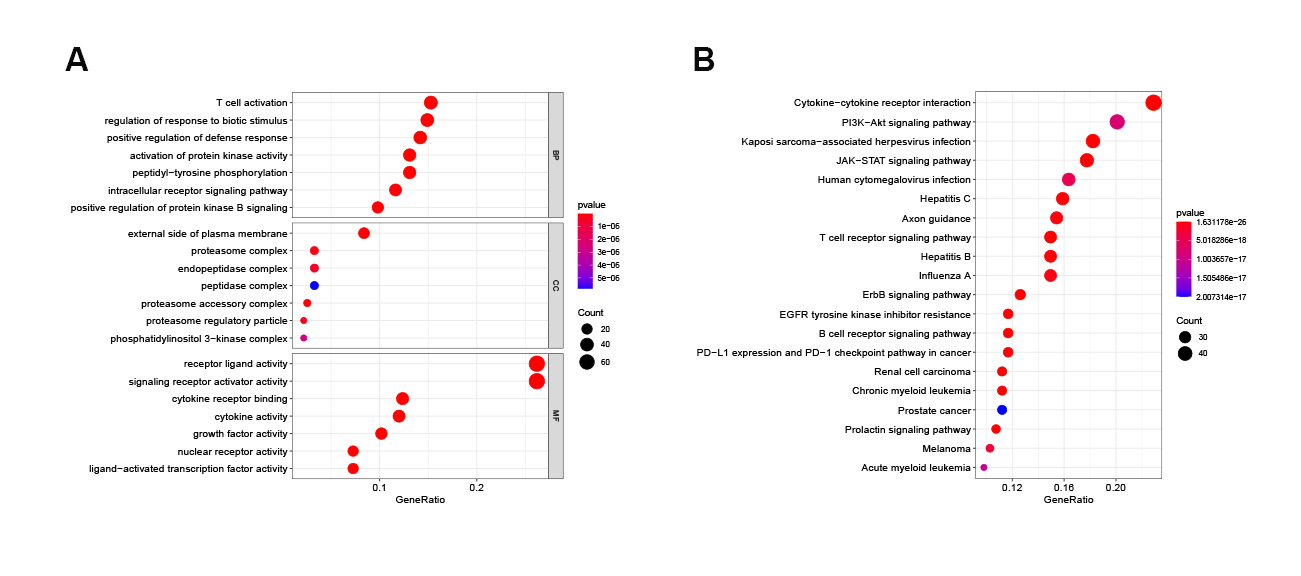

Supplement: Supplementary 1 — Gene enrichment analysis of m6A-related immune genes. (A) Bubble plot of GO analysis. (B) Bubble plot of KEGG analysis. [file DataSheet_1.zip › Supplementary 1.JPEG]

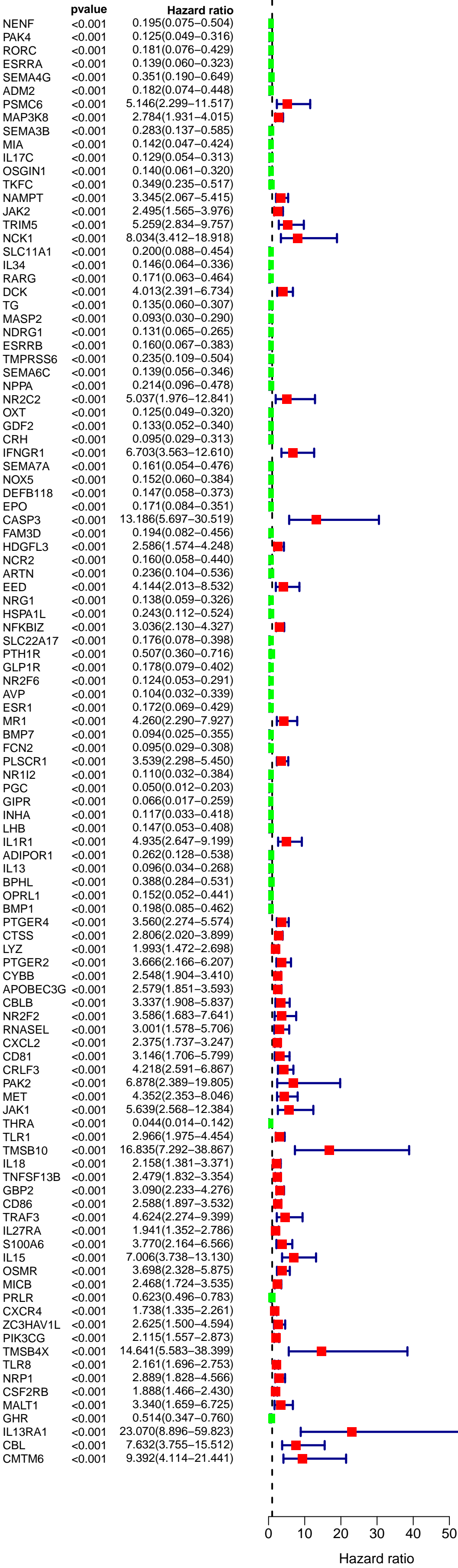

Supplement: Supplementary 1 — Gene enrichment analysis of m6A-related immune genes. (A) Bubble plot of GO analysis. (B) Bubble plot of KEGG analysis. [file DataSheet_1.zip › Supplementary 2.PDF]

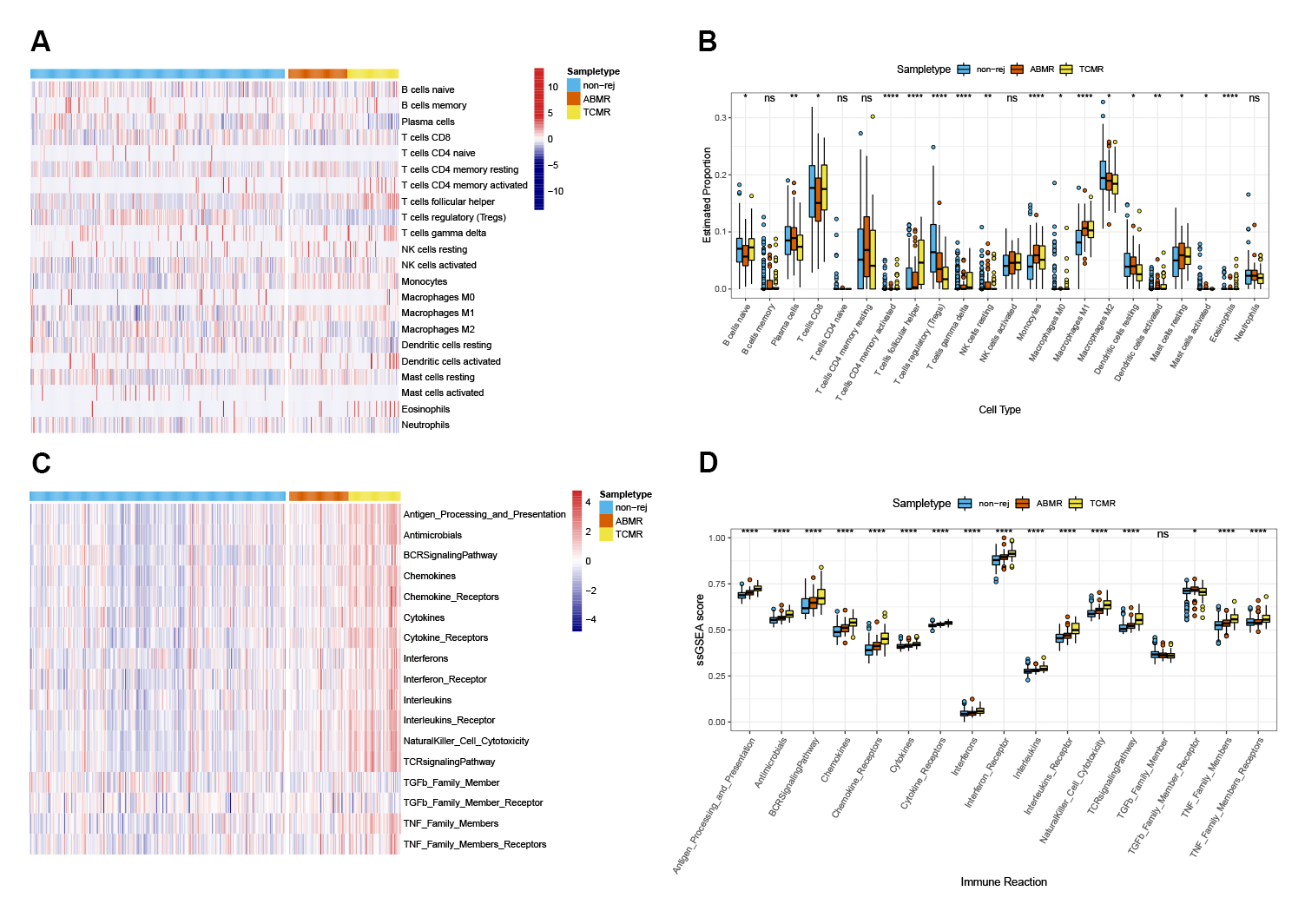

Supplement: Supplementary 1 — Gene enrichment analysis of m6A-related immune genes. (A) Bubble plot of GO analysis. (B) Bubble plot of KEGG analysis. [file DataSheet_1.zip › Supplementary 3.JPEG]

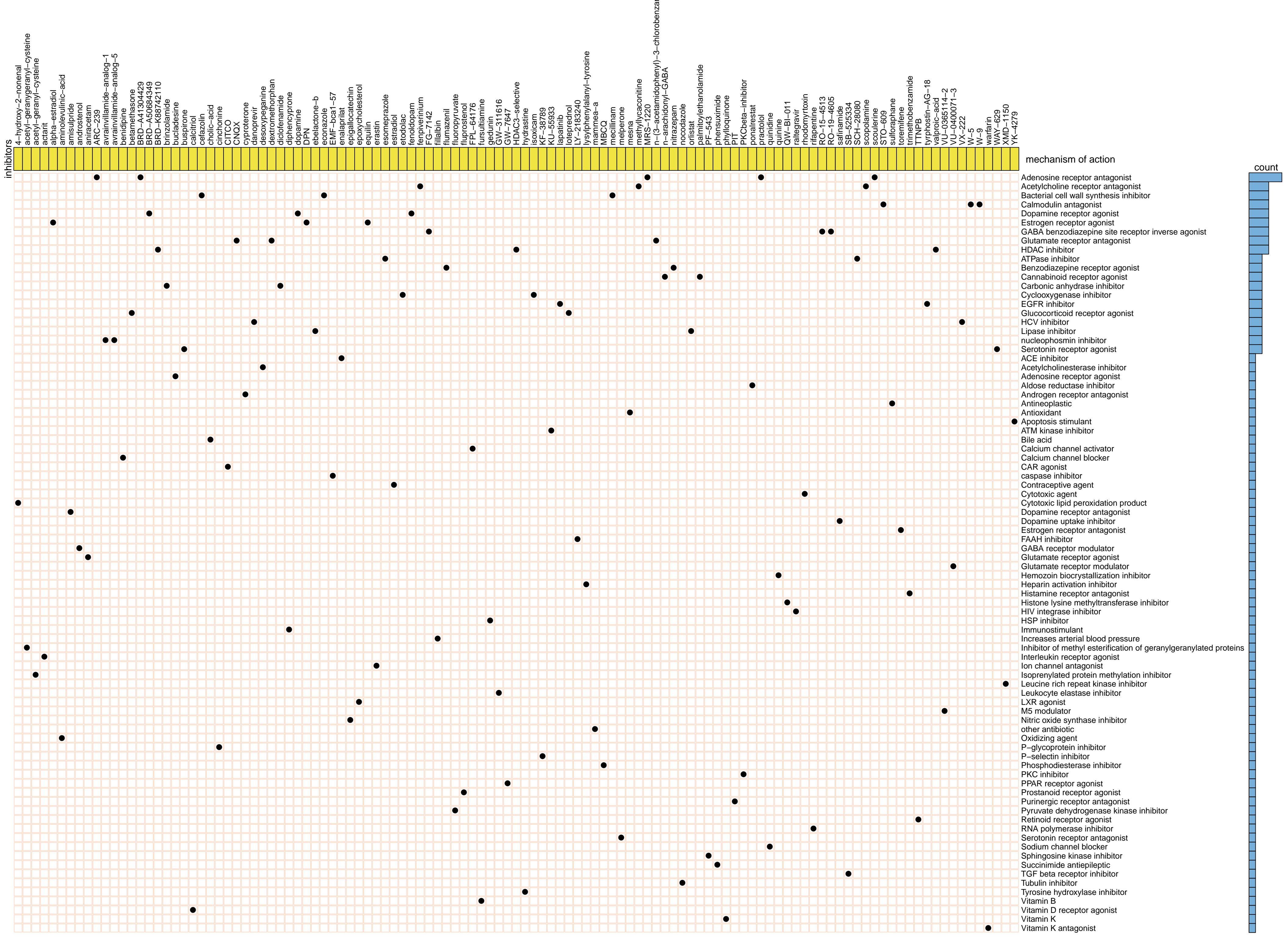

Supplement: Supplementary 1 — Gene enrichment analysis of m6A-related immune genes. (A) Bubble plot of GO analysis. (B) Bubble plot of KEGG analysis. [file DataSheet_1.zip › Supplementary 5.PDF]
